# Supplementary material for: Genome-wide CRISPR Screens in T Helper Cells Reveal Pervasive Crosstalk between Activation and Differentiation
Source: Cell. 2019 Feb 7;176(4):882–896.e18. doi: 10.1016/j.cell.2018.11.044 (PMC6370901; doi:10.1016/j.cell.2018.11.044)
Supplement: Data S2. Processed Data from All the Steps of the Analysis, Related to Figure 1 [file mmc2.zip › supplemental data/motif analysis/Pparg_homer/homerResults/motif1.similar.html]

motif1

## Information for motif1

C
A
G
T
T
C
G
A
A
C
G
T
A
C
T
G
C
G
T
A
A
T
C
G
C
G
A
T
T
G
A
C
C
G
T
A
A
G
C
T
  
Reverse Opposite:  

T
C
G
A
C
G
A
T
A
C
T
G
C
G
T
A
A
T
G
C
A
C
G
T
T
G
A
C
C
G
T
A
A
G
C
T
G
T
C
A
  

|  |  |
| --- | --- |
| p-value: | 1e-1923 |
| log p-value: | -4.430e+03 |
| Information Content per bp: | 1.684 |
| Number of Target Sequences with motif | 5063.0 |
| Percentage of Target Sequences with motif | 40.44% |
| Number of Background Sequences with motif | 3241.6 |
| Percentage of Background Sequences with motif | 9.05% |
| Average Position of motif in Targets | 101.8 +/- 51.7bp |
| Average Position of motif in Background | 101.6 +/- 68.5bp |
| Strand Bias (log2 ratio + to - strand density) | -0.0 |
| Multiplicity (# of sites on avg that occur together) | 1.24 |
| Motif File: | file (matrix) reverse opposite |

### Similar de novo motifs found

|  |  |  |  |  |  |  |  |
| --- | --- | --- | --- | --- | --- | --- | --- |
| Rank | Match Score | Redundant Motif | P-value | log P-value | % of Targets | % of Background | Motif file |
| 1 | 0.989 | T C G A C G A T A C T G C G T A A T C G C G A T T G A C C G T A A C G T G C T A G C A T G C A T | 1e-1820 | -4191.671552 | 42.66% | 10.74% | motif file (matrix) |
| 2 | 0.982 | T A C G A G C T A C T G G T C A A T G C C G A T G T A C C G T A | 1e-1810 | -4169.763234 | 43.70% | 11.38% | motif file (matrix) |
| 3 | 0.941 | T C G A G A C T A C T G G C T A T A G C C G A T T A G C C G T A A C G T G T A C | 1e-1109 | -2555.561883 | 55.96% | 25.79% | motif file (matrix) |
| 4 | 0.744 | C G T A A T C G C G A T T G A C C G T A G A C T G C T A A G C T G A T C C T A G | 1e-836 | -1926.556973 | 46.14% | 21.22% | motif file (matrix) |
| 5 | 0.758 | C A G T C G T A G A C T A T C G C G T A G T A C C G A T T A G C | 1e-692 | -1594.871072 | 76.01% | 51.50% | motif file (matrix) |
| 6 | 0.801 | C G A T T C A G G A T C G A C T C G A T G C T A T A C G A C G T T G A C C G T A | 1e-578 | -1332.944277 | 29.46% | 12.15% | motif file (matrix) |
| 7 | 0.728 | C T G A A T G C C G A T G T A C C G T A A G C T G A C T G A C T | 1e-555 | -1278.347820 | 26.23% | 10.26% | motif file (matrix) |
| 8 | 0.662 | G A C T G T C A A T G C C G T A A T C G A G C T A G T C C G T A A G C T G A C T G A C T A G T C | 1e-528 | -1217.607281 | 23.99% | 9.08% | motif file (matrix) |
| 9 | 0.722 | A G C T C A G T G T C A A G T C G A C T G T C A T G C A A C T G | 1e-522 | -1202.360269 | 19.31% | 6.30% | motif file (matrix) |
| 10 | 0.676 | T A C G C G A T C T G A C T A G A C T G A G C T A T C G C G T A | 1e-488 | -1125.795020 | 33.73% | 16.38% | motif file (matrix) |
| 11 | 0.605 | T A C G G C A T A C T G G T C A G A C T T C A G C G A T A T C G G A C T A G T C G T C A G A C T | 1e-196 | -452.332726 | 17.24% | 8.78% | motif file (matrix) |
| 12 | 0.609 | G A C T G A C T G A C T G C T A G A T C G C A T T A G C C G T A T A C G G T C A T G C A T C G A | 1e-103 | -237.516953 | 15.07% | 9.06% | motif file (matrix) |
| 13 | 0.644 | C A T G G C A T T C G A G A T C C G T A T A C G G C A T G T A C C T G A C G A T | 1e-88 | -204.609944 | 2.76% | 0.75% | motif file (matrix) |
